# Weight Loss in Nonalcoholic Fatty Liver Disease Patients in an Ambulatory Care Setting Is Largely Unsuccessful but Correlates with Frequency of Clinic Visits

**DOI:** 10.1371/journal.pone.0111808

**Published:** 2014-11-06

**Authors:** Anwar Dudekula, Vikrant Rachakonda, Beebijan Shaik, Jaideep Behari

**Affiliations:** 1 Department of Medicine, Division of General Internal Medicine, University of Pittsburgh School of Medicine, Pittsburgh, Pennsylvania, United States of America; 2 Department of Medicine, Division of Gastroenterology, Hepatology, and Nutrition, University of Pittsburgh School of Medicine, Pittsburgh, Pennsylvania, United States of America; Institute of Medical Research A Lanari-IDIM, University of Buenos Aires-National Council of Scientific and Technological Research (CONICET), Argentina

## Abstract

**Background and Aims:**

Nonalcoholic fatty liver disease (NALFD) is a leading cause of liver disease. Weight loss improves clinical features of NAFLD; however, maintenance of weight loss outside of investigational protocols is poor. The goals of this study were to characterize patterns and clinical predictors of long-term weight loss in ambulatory patients with NAFLD.

**Methods:**

We retrospectively reviewed 924 non-cirrhotic patients with NAFLD presenting to a liver clinic from May 1^st^ 2007 to April 30^th^ 2013. Overweight and obese patients were counseled on lifestyle modifications for weight loss as per USPSTF guidelines. The primary outcome was percent weight change between the first and last recorded visits: % weight change  =  (weight^initial^ – weight^final^)/(weight^initial^). Baseline BMI and percent BMI change were secondary measures. Predictors of weight loss were determined using logistic regression.

**Results:**

The mean baseline BMI was 33.3±6.6 kg/m^2^, and the mean follow-up duration was 17.3±17.6 months. Most patients with NAFLD were in either overweight (26.1%) or class I obesity (30.5%) categories at baseline, while the prevalence of underweight and class III obesity was lower (0.2% and 15.4%, respectively). Overall, there was no change in mean weight or BMI during the follow-up period, and only 183 patients (19.8%) lost at least 5% body weight during the follow up period. Independent predictors of weight loss included number of clinic visits and baseline BMI, and patients with higher baseline BMI required more clinic visits to lose weight.

**Conclusions:**

Weight loss is largely unsuccessful in NAFLD patients in the ambulatory care setting. Frequent clinical encounters are associated with weight reduction, especially among individuals with high baseline BMI. Future studies are required to define effective weight loss strategies in NAFLD patients.

## Introduction

With the rise of obesity and the metabolic syndrome, nonalcoholic fatty liver disease (NAFLD) has become a major risk factor for both cardiovascular disease and cirrhosis [1]. By conservative estimates, approximately 20–30% of adults in developed nations are affected. In the United States, population based studies have reported a prevalence of 10–46%, while the prevalence of biopsy-proven nonalcoholic steatohepatitis (NASH) ranges from 3–5% [2]–[4]. NAFLD is also an emerging health concern in the developing world; as up to 30% of certain Asian populations exhibit significant steatosis [5]. Although age, hypertension, gender, diet and genetic polymorphisms have been implicated as predictors for the development of NAFLD, the strongest risk factors are insulin resistance and obesity. In addition, it has been suggested that NAFLD rates rise with increasing levels of obesity [2], [6].

Multiple trials have demonstrated that weight loss reduces intrahepatic fat content and improves serum aminotransferases [7]–[11]. Furthermore, increased weight loss is associated with greater improvement in histologic steatosis, hepatocyte ballooning, and lobular inflammation [8], [10]. Based on these findings, practice guidelines from the American Association for the Study of Liver Diseases recommend weight loss as a treatment for NAFLD [12]. The optimal approach to weight loss, however, remains unclear, as multiple interventions, including dietary modification, physical activity, medications, and bariatric surgery are currently used. Additionally, maintenance of weight loss outside of investigational protocols remains challenging. In a pooled follow-up analysis of three large weight loss trials, 28% of patients maintained weight loss in the second year after intervention, while only 23% did not gain weight in the third year [13]. These findings and others highlight concerns regarding effective implementation of investigational weight loss protocols in real-world practice settings.

Currently, American Association for the Study of Liver Diseases (AASLD) guidelines suggest that weight loss of 3–5% may improve hepatic steatosis, while weight loss of 10% or more may be required to improve necroinflammation [12]. The effectiveness of these weight loss recommendations in NAFLD patients outside of clinical trials is not known. Therefore, the goal of this analysis was to characterize patterns of long-term weight loss in a large, ambulatory cohort of patients with NAFLD. In addition, we sought to determine clinical predictors of successful weight loss in this population.

## Methods

### Ethics statement

The University of Pittsburgh institutional Review Board approved this retrospective cohort study with a waiver of consent (approval number PRO12030073). Subjects with NAFLD were identified by ICD-9 codes using the University of Pittsburgh Center for Liver Diseases (CLD) Research Registry, which consists of ambulatory patients with chronic liver diseases presenting to the CLD for long-term care. All patient records were de-identified prior to analysis.

### Study design and participants

A flow diagram of the population selection strategy is depicted in **Figure S1.** Patients initially encountered in the CLD from May 1^st^ 2007 to April 30^th^ 2013 were included in the study. Subjects 18 years of age or older with NAFLD were identified from the registry using ICD-9-CM code 571.8 (“other chronic nonalcoholic liver disease”), and those with two or more subsequent visits were included in the analysis. Individuals were excluded if documented height or weight measurements were missing at initial or subsequent encounters. Patients with other liver diseases (determined by their ICD9 codes) including viral hepatitis, autoimmune hepatitis, hemochromatosis and alpha-one antitrypsin deficiency were also removed. Other exclusion criteria included human immunodeficiency virus (HIV), celiac disease, prior solid organ transplant, hepatocellular carcinoma, prior gastrointestinal bypass surgery and ongoing ethanol use. Initially, patients with cirrhosis were excluded, as previous studies have demonstrated that total body weight does not accurately reflect body composition in this population [14], [15]. In particular, cirrhotic subjects with ascites are prone to iatrogenic weight loss due to diuretic use or large volume paracentesis. A previously validated combination of ICD-9 codes was used to identify cirrhotic patients among the NAFLD cohort [16], and these were removed from the primary analysis. We then also assessed the primary outcome (percentage weight change) after including cirrhotic subjects. Patients with two or more clinic visits, with documented heights and weights were included for analysis. We used the same exclusion criteria used for pure NAFLD patients, such as viral hepatitis, autoimmune hepatitis, alpha-one antitrypsin deficiency, human immunodeficiency virus (HIV), celiac disease, prior solid organ transplant, hepatocellular carcinoma, prior gastrointestinal bypass surgery and ongoing ethanol use. The primary outcome, percent weight change was assessed exactly as for noncirrhotic NAFLD patients.

Subjects were followed from the first clinical encounter on or after May 1^st^ 2007 until their final clinical encounter on or before April 30^th^, 2013. The primary outcome was percent weight change, which was measured between the first and last recorded visits: % weight change  =  (weight^initial^ – weight^final^)/(weight^initial^). Weight change was then classified into 5 categories: weight gain >10%, weight gain 5–10%, weight change <5%, weight loss 5–10%, and weight loss >10%. Secondary outcomes included absolute and percent change in BMI (absolute BMI change  =  BMI^intial^ – BMI^final^; % BMI change  =  (BMI^initial^ – BMI^final^)/BMI^initial^).

### Clinical and demographic variables

All patient encounters took place in a single climate controlled, indoor clinic, and all subject wore light clothing without shoes for measurements. Patient age and gender were recorded; patient race was self-reported. Three weight measurements were obtained by trained medical assistants using a calibrated beam balance scale, and two consistent results were recorded. Among these, one was chosen as the final weight; and this result was recorded to the nearest 0.1 kg. Height was measured to the nearest centimeter using a stadiometer positioned at the top of each patient's head.

Body mass index (BMI) was determined at the initial and final visits using the following formula: BMI  =  mass (in kg)/(height in m^2^). Baseline BMI was stratified according to World Health Organization (WHO) definitions of obesity as follows [17]: underweight (<18.5 kg/m^2^), normal weight (18.5–24.99 kg/m^2^), overweight (25.0–29.99 kg/m^2^), class I obesity (30.0–34.99 kg/m^2^), class II obesity (35.0–39.99 kg/m^2^), and class III obesity (≥40 kg/m^2^).

The presence of comorbid conditions associated with obesity was abstracted from the Registry database using associated ICD-9 codes. These included diabetes mellitus (DM), hypertension, hyperlipidemia, gastrointestinal reflux disease (GERD) and psychiatric conditions such as depression and anxiety. The follow-up interval was calculated as follows: [(date of last visit) – (date of first visit)]/30 and expressed in units of months. Values were rounded to one decimal place. The number of visits per years was defined as follows: number of total visits (n)/follow-up duration (years).

During each clinic visit, patients were counseled by their physician or advanced practice provider regarding the importance of weight loss for reducing liver fat and liver inflammation. Providers provided counseling to their patients during the office visit about the health benefits of weight loss and lifestyle changes as outlined in US Preventive Services Task Force (USPSTF) guidelines [18], [19]. Since the purpose of this study was to determine the effectiveness of routine medical care outside of a clinical trial to encourage weight loss in patients with NAFLD, the providers in the practice were not required to adhere to standardized recommendations for lifestyle modifications.

### Statistical analysis

Statistical analysis was performed with Stata version 12 (StataCorp, College Station, TX). Continuous variables are presented as mean ± standard deviation (S.D.) or median (interquartile range [IQR]) for parametric and non-parametric data respectively, and categorical variables are presented as absolute frequencies and percentages. Normality of the continuous variables was assessed visually with histograms. Comparisons between groups were performed with the χ^2^ test for categorical variables (or Fischer's exact test when expected values were ≤5), while the student's t test was used for continuous variables. Logistic regression analysis was used to determine predictors of weight loss of at least 5%, which was coded as a dichotomous variable. Individuals who achieved weight loss of 5% or more were coded “1”, while those who did noted were coded as “0”. Patient characteristics with *p*<0.25 in the univariate analysis were then included in multivariable model. Collinearity was assessed by variance inflation factor calculation and pairwise correlation; Akike Information Criterion analysis was then used to compare models excluding each collinear variable separately to determine which to exclude from the final multivariable model. A *p* value (two-tailed) less than or equal to 0.05 was considered statistically significant.

As physicians may schedule frequent return clinic visits for patients with higher BMI, an interaction term between number of clinic visits and baseline BMI was included in the multivariable logistic regression model, and results were interpreted using adjusted marginal effects methods [20].

## Results

### Clinical and demographic features of patients

Clinical and demographic characteristics are summarized in **Table 1** **.** The study cohort included 924 subjects (Table S1). The population was predominantly Caucasian (n = 870, 94.2%) and female (n = 552, 59.7%). The median duration of follow-up was11 months (IQR 3.5–22.5 months), and on average, 3.8±2.5 visits occurred during that interval. Overall, the cohort was obese, and the mean weight of the patients at the time of the first visit was not significantly different than the mean weight of the patients at the time of last clinic visit. Similarly, the mean BMI of the patient population at the time of the first visit was not significant different than the mean BMI of the patients at the time of last clinic visit. Comorbidities associated with obesity such as hypertension, diabetes mellitus, and GERD were commonly encountered in the study population, and 10% of patients suffered from psychiatric illness. Six individuals (0.7%) died during the study.

**Table 1:** Demographics and clinical characteristics of the study population (N = 924).

VARIABLE	
Age (years)*	53.6±12.7
Duration of follow up (months)*	11(3.5,25.5)
Number of clinic visits*	3.8±2.5
Average number of visits per year*	1.7±0.6
Initial BMI (kg/m^2^)*	33.3±6.6
Final BMI (kg/m^2^)*	33±6.6
Initial Weight (kg)*	95.1±20.8
Final Weight (kg)*	94.4±20.7
Male gender^†^	372 (40.3%)
Race^†^	
White	870 (94.2%)
African-American	27 (2.9%)
Others	27 (2.9%)
Hypertension^†^	191 (20.7%)
Diabetes mellitus^†^	163 (17.6%)
Psychiatric comorbidity^†^	92 (10%)
Hyperlipidemia^†^	253 (27.4%)
Death^†^	6 (0.7%)

Values presented as *mean ± SD or ^†^N (%) or median(ICR).

**Figure 1:**
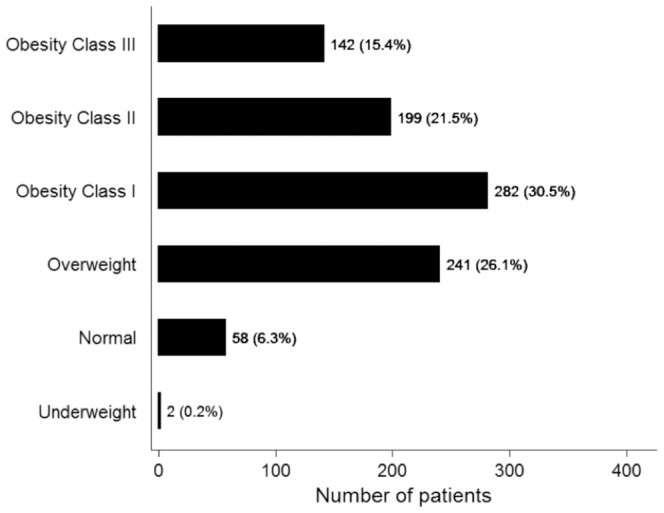
Initial body mass index (BMI) distribution of 924 non-cirrhotic NAFLD patients. Baseline BMI was stratified using the following definitions: underweight (<18.5 kg/m^2^), normal weight (18.5–24.99 kg/m^2^), overweight (25.0–29.99 kg/m^2^), class I obesity (30.0–34.99 kg/m^2^), class II obesity (35.0–39.99 kg/m^2^), and class III obesity (≥40 kg/m^2^).

The baseline BMI profile is depicted in **Figure 1**, and a bell-shaped distribution was observed. The majority of patients with NAFLD had either overweight (26.1%) or class I obesity (30.5%), while the prevalence of underweight and class III obesity was significantly lower.

### Patterns of Weight Change and Predictors of Weight Loss in NAFLD

**Figure 2:**
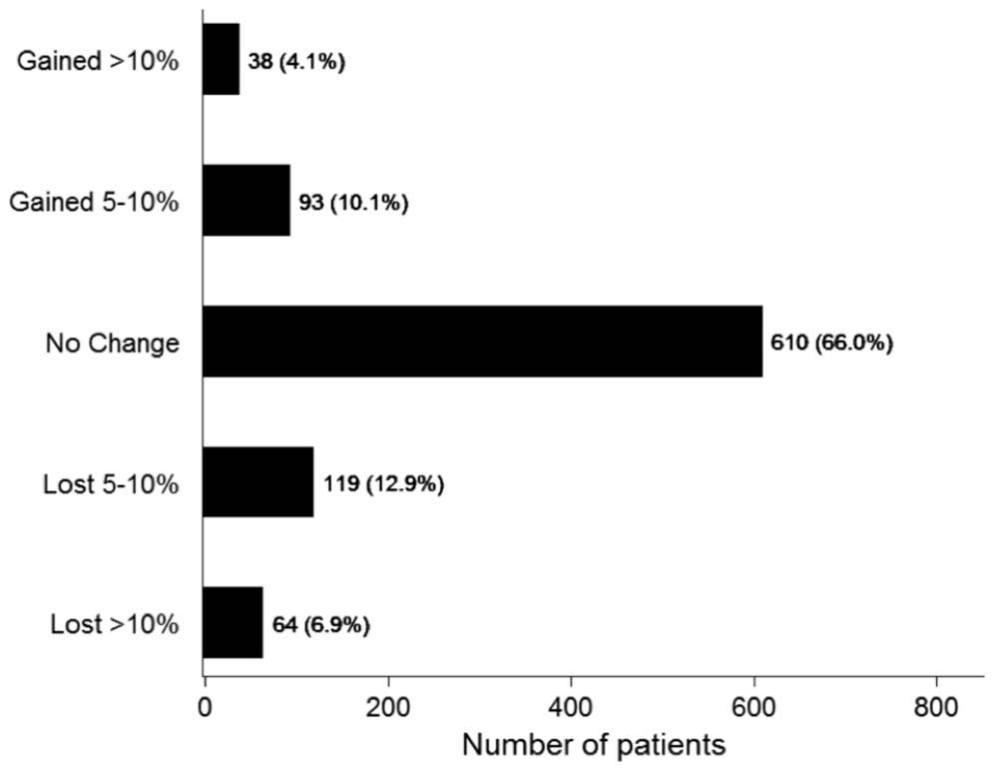
Percent change in body weight at the end of the study period relative to the initial weight. Patients with final body weight within 5% of their initial weight were included in the “No change” group. N = 924 for the entire cohort.

We characterized patterns of weight change in our NAFLD cohort, and these findings are depicted in **Figure 2**. The majority of patients experienced minimal weight change of less than 5% during the study period (n = 610, 66%); however, weight loss of at least 5% was more common than weight gain of at least 5% in this cohort (183 versus 131 patients). NAFLD can lead to the development of liver cirrhosis and many patients have established cirrhosis when they are diagnosed. Our initial analysis was limited to NAFLD patients without established cirrhosis. Therefore, we next examined weight loss in an expanded cohort consisting of 1413 NAFLD patients with and without cirrhosis. The results were similar to that in noncirrhotic NAFLD patients, with the majority of individuals experiencing weight change of less than 5% (data not shown).

Next, logistic regression methods were used to determine predictors of weight loss of 5% or more (**Tables 2** **and** **3**). While duration of follow-up was identified as a predictor of weight loss in the univariate analysis, it was not significantly associated with weight loss in the multivariate model. The main effects of initial BMI and the total number of clinic visits in the model cannot be interpreted in the presence of a significant interaction (*p* = 0.05). The odds ratio of weight loss of 5% or more for one unit increase in initial BMI at two, three, four and five (or more) clinic visits was 0.98 (*p* = 0.68), 1.04 (*p* = 0.11), 1.05 (*p* = 0.09) and 1.10 (*p*<0.001) respectively. This demonstrates that initial BMI was significantly associated with weight loss of 5% or more only for those patients with five or more clinic visits; among this subset, increased BMI is associated with increased odds of weight loss of 5% or more.

**Table 2:** Univariate logistic regression for predictors of weight loss (N = 924).

Variable	OR	95% CI	P value
Duration of follow up	1.01	1.004,1.02	**0.003**
Age	1.00	0.99,1.02	0.70
Average number of visits per year	1.19	0.90,1.56	0.22
Number of distinct years seen in clinic			
1 year	Baseline		**<0.001**
2 year	2.08	1.33,3.24	
3 years	1.93	1.11,3.38	
≥4 years	2.70	1.62,4.49	
Total Number of Visits			
2 visits	Baseline		**<0.001**
3 visits	3.26	2.03,5.24	
4 visits	3.74	2.23,6.30	
≥5 visits	3.29	1.89,5.75	
Diabetes mellitus	1.29	0.86,1.94	0.22
Hyperlipidemia	1.34	0.94,1.90	0.1
Initial BMI*Total Number of Clinic Visits			0.045
2 visits	Baseline		
3 visits	1.055	.99,1.14	
4 visits	1.063	0.98.1.15	
5 visits	1.11	1.02,1.19	
Gender			
Male	Baseline		0.34
Female	1.18	0.84,1.64	
Psychiatric Comorbidity	0.7	0.39,1.27	0.25
Hypertension	0.93	0.62,1.39	0.71
Initial BMI	1.03	1.02,1.06	**0.001**
Race			
Caucasian	Baseline		0.75
African-American	0.7	0.24,2.05	
Other	1.15	0.46,2.89	

Abbreviations: OR, odds ratio; CI, confidence interval; BMI, body mass index.

**Table 3:** Multivariate logistic regression for predictors of weight loss (N = 924).

Variable	Model With Interaction			Model Without Interaction		
	AOR	95% CI	P value	AOR	95% CI	P value
Total number of visits						
2 visits	Baseline		**0.29**	Baseline		<0.001
3 visits	0.55	0.04,7.01		3.43	2.03,5.79	
4 visits	0.49	0.03,7.57		3.88	2.09,7.19	
≥5 visits	0.09	0.01,1.21		3.37	1.58,7.16	
Duration of follow up	0.99	0.97,1.02	0.66	0.99	0.97,1.01	0.6
Initial BMI	0.99	0.94,1.04	<0.001	1.05	1.02,1.07	0.004
Number of distinct years seen in clinic						
1 year	Baseline		0.28	Baseline		0.27
2 year	1.23	0.73,2.08		1.32	0.78,2.23	
3 years	0.81	0.37,1.78		0.89	0.41,1.94	
≥4 years	1.38	0.45,4.19		1.49	0.49,4.51	
Hyperlipidemia	1.39	0.93,2.06	0.45	1.33	0.89,1.97	0.16
Initial BMI*Total Number of Clinic Visits						
2 visits	Baseline		**0.04**			
3 visits	1.06	0.98,1.14				
4 visits	1.06	0.98,1.15				
5 visits	1.11	1.03,1.19				

Abbreviations: AOR, Adjusted odds ratio; CI, confidence interval; BMI, body mass index.

To increase comprehension/applicability of the model, we also assessed odds ratio of weight loss of 5% or more for a patient with average BMI (33.3 kg/m^2^) for three 3.33 (*p*<0.001), four 3.78 (*p*<0.001) and five (or more) 3.08 (*p* = 0.004) clinic visits compared to those with two clinic visits respectively.

**Figure 3:**
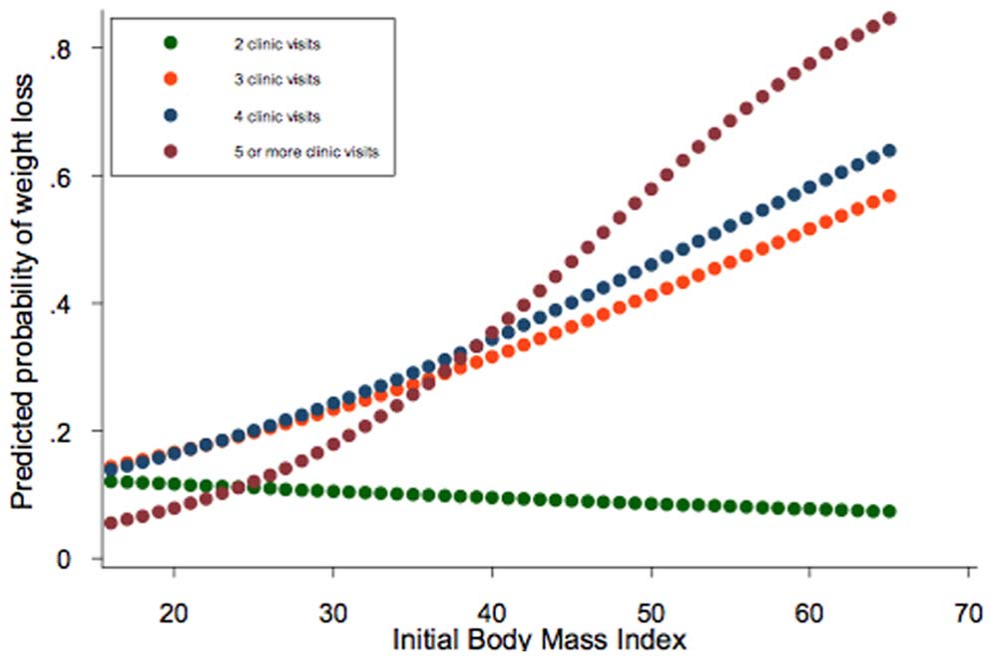
Predicted probability of weight loss (>5% of initial weight) based on number of clinic visits and BMI. Patients were stratified based on their initial BMI (used as a continuous variable) and number of clinic visits. N = 924 total patients; 2 visits: 354; 3 visits: 202; 4 visits: 130; and 5 or more visits: 238. P<0.001 for 2 visits versus 3, P<0.05 for 2 visits versus 4, and P =  NS for 2 versus 5 or more visits. P =  NS for 3 versus 4, or 5 or more visits.

The cumulative effect of initial BMI and clinic visits on weight loss probability is depicted in **Figure 3**. In patients with at least three clinic visits, the probability of weight loss of 5% or more increased proportionally with rising baseline BMI, while among those with two encounters, the probability of weight loss decreased with higher baseline BMI.

## Discussion

Four novel observations were made in this study, which characterizes patterns of weight change in a large ambulatory cohort with NAFLD. First, most non-cirrhotic patients with NAFLD exhibited either overweight (BMI 25.0–29.99 kg/m^2^) or Class I obesity (BMI 30.00–34.99 kg/m^2^), while the prevalence of Class II or III obesity was lower. Second, over 80% of patients failed to lose at least 5% body weight during the follow-up period, and this finding was reproduced in a larger cohort of both cirrhotic and non-cirrhotic NAFLD patients. Third, we identified baseline BMI and number of clinical visits as independent predictors of weight loss. Last, but not the least, weight loss in real life setting in outpatient clinics is largely unsuccessful.

Obesity is a primary risk factor for the development of NAFLD [2], [12], and the prevalence of NAFLD rises with increasing BMI and is associated with development of cirrhosis [21], [22]. In the current study, however, the majority of patients had either overweight or class I obesity, while fewer patients exhibited more advanced obesity. Weight loss is an effective treatment for NAFLD, as trials of both lifestyle modification and pharmacologic weight reduction have demonstrated histologic improvements in both steatosis and inflammation with 5% or more weight loss [8], [10].

In this study, we found that baseline BMI and number of clinic visits were independently associated with significant weight loss, while duration of follow-up was not a significant predictor. The effect of baseline BMI on weight loss may be related to altered energy expenditure. As increased baseline BMI is associated with heightened basal metabolic rate, weight loss programs utilizing caloric deficits may preferentially enhance early weight loss in this population [23], [24]. Although it is well-documented that a combination of educational, behavioral and motivational strategies help patients achieve healthy weight loss [25], [26], maintenance of weight remains challenging. Nonetheless, there is evidence that frequent clinical encounters preserve weight loss. In a trial comparing face-to-face visits, quarterly newsletters, and Internet-based interactions for maintenance of weight loss, subjects who underwent clinical encounters gained less weight than those in nonpersonal intervention groups [27]. Comparable results were observed in a subsequent analysis comparing personal encounters to non-human interventions for weight loss maintenance [28]. Consistent with these findings, we observed that patients with three or more clinical visits had a significantly increased probability of losing weight. This effect may be related to increased patient exposure to counseling regarding weight loss with more frequent clinical visits, and future studies are planned to test this hypothesis.

To assess the relationship between initial BMI and number of clinic visits on weight loss, we modified the original multivariable regression model to include an interaction between these predictors. Two important findings were noted. First, weight loss probability increased proportionally with baseline BMI in patients with three or more visits, while it decreased inversely with BMI in those with two visits, suggesting that two visits may be insufficient for weight loss in severely obese individuals. Second, the probability of weight loss was higher in patients with three or more visits compared to those with two encounters. The advantage of additional clinic visits, however, was dependent on baseline BMI, as more encounters were required to confer a benefit in patients with higher baseline BMI. Together, these findings underscore the importance of frequent clinical interactions across multiple BMI levels.

Our results have clinical implications for practitioners taking care of patients with nonalcoholic fatty liver disease. There is an urgent need to define practical and effective interventions to promote weight loss in this population. Most encounters in our cohort were one-to-one interactions between patients and providers, which our results suggest are largely unsuccessful. Thus, an alternate multidisciplinary approach, which includes evaluation and advice provided by dieticians, exercise physiologists, and, perhaps, psychologist, is likely to be beneficial, although a major barrier to this multidisciplinary approach in the US is lack of insurance coverage for these services.

Our results suggest that individual providers can have an impact on the achievement of weight loss in their patients through regular and frequent counseling during face-to-face encounters. It is also important to emphasize that even modest weight loss can have significant long-lasting effects on improvement in liver steatosis, even if patients later regain weight, and that exercise is a promising intervention for NAFLD even in the absence of weight loss [29]–[31].

A few limitations of this study should be noted. First, this is a retrospective analysis. However, data for a large number of patients was accumulated for an extended duration, and by examining an ambulatory cohort, we were able to capture features of weight loss in a real-world clinical practice. Second, weight loss interventions were limited to brief counseling according to USPSTF guidelines [18], [19], and it would useful to study particular techniques utilized by those who lost significant weight. Moreover, as only initial and final weight data were available for anaylsis, temporal fluctuations in weight were not recorded. Next, information about food intake and physical activity were not obtained, and both of these factors may influence intrahepatic fat content independent of changes in body weight [30], [31]. Finally, there was significant variability in follow-up intervals, but this was accounted for in multivariate logistic regression models. Neither number of visits per year nor follow-up duration signficantly influenced the odds of weight loss.

In conclusion, our findings demonstrate that among patients with NAFLD, weight loss is largely unsuccessful in real world clinical settings. All the same, there appears to be a beneficial impact from clinician interactions, as increased number of clinical encounters is associated with enhanced probability of weight reduction. Future studies are required to target successful weight loss strategies to high-risk populations.

## Supporting Information

Figure S1 Flow diagram showing an overview of the strategy used to identify all adult non-cirrhotic patients with NAFLD. The ICD-9 571.8 is for “Other chronic nonalcoholic liver disease” and includes NAFLD and NASH.
(TIF)

Table S1 Relevant clinical data associated with the 924 patients included in the study.
(XLSX)
