# Supplementary figures and images for: Weight Loss in Nonalcoholic Fatty Liver Disease Patients in an Ambulatory Care Setting Is Largely Unsuccessful but Correlates with Frequency of Clinic Visits
Source: PLoS One. 2014 Nov 6;9(11):e111808. doi: 10.1371/journal.pone.0111808 (PMC4222918; doi:10.1371/journal.pone.0111808)

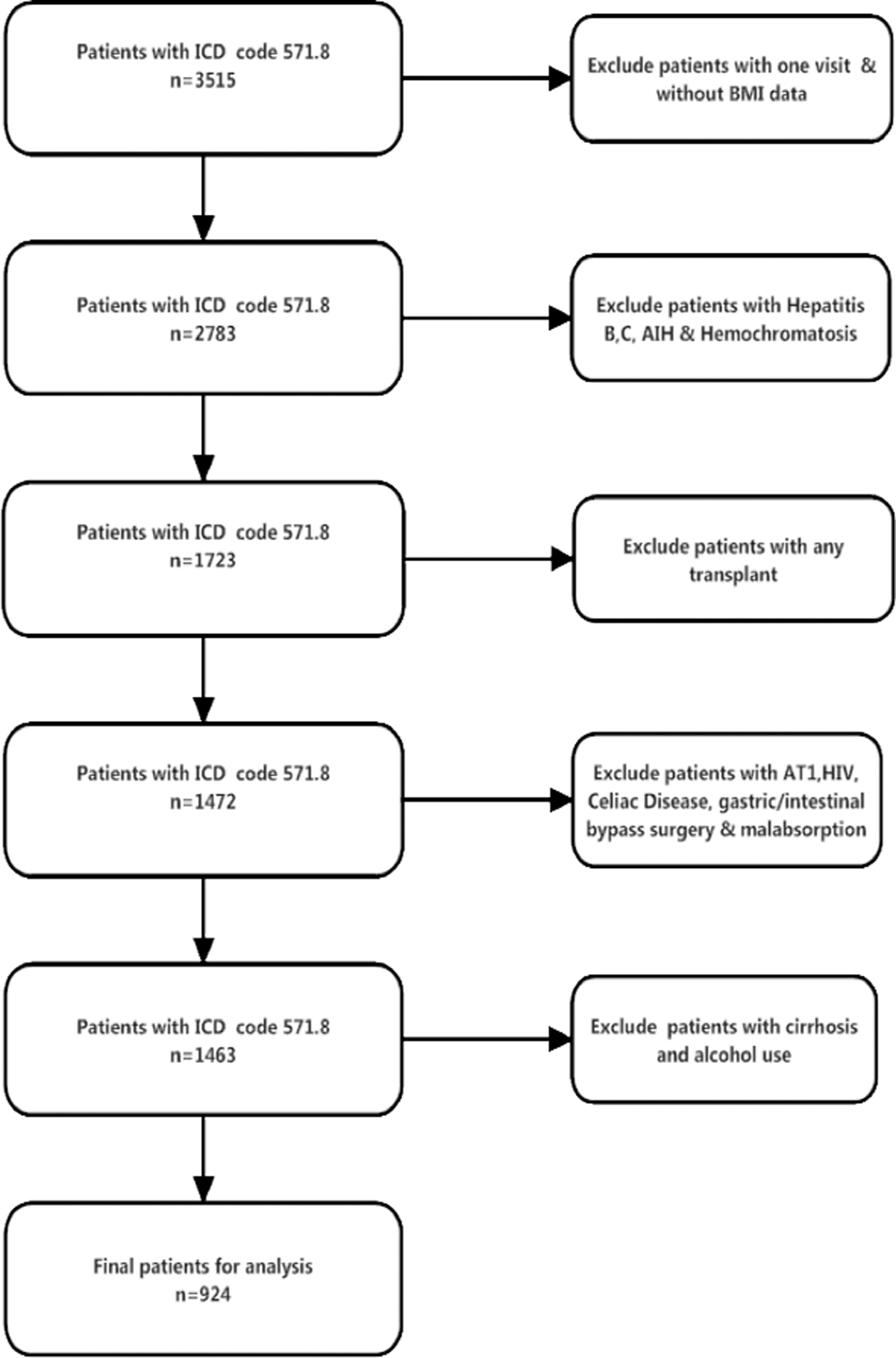

Supplement: Figure S1 — Flow diagram showing an overview of the strategy used to identify all adult non-cirrhotic patients with NAFLD. The ICD-9 571.8 is for “Other chronic nonalcoholic liver disease” and includes NAFLD and NASH. (TIF) [file pone.0111808.s001.tif]
